# Pomalidomide-induced lung injury: A case report

**DOI:** 10.1097/MD.0000000000032473

**Published:** 2023-01-13

**Authors:** Alexandre Vivien, Julien Ancel, Sophie Godet, Sandra Dury, Jeanne-Marie Perotin, Gaetan Deslee, Claire Launois

**Affiliations:** a Department of Respiratory Diseases, Reims University Hospital, Reims, France; b Department of Hematology, Reims University Hospital, Reims, France; c INSERM UMR-S 1250 “Pathologies Pulmonaires Et Plasticité Cellulaire,” University of Reims Champagne-Ardenne, Reims, France.

**Keywords:** lung injury, multiple myeloma, pomalidomide, side effect

## Abstract

Pomalidomide is an immunomodulatory imide drug used in multiple myeloma and in Kaposi sarcoma.

**Patient concerns:**

A 72-years-old male, treated for multiple myeloma with dexamethasone, pomalidomide and daratumumab, presented dyspnea, hypoxemia, biological inflammatory syndrome, ground glass opacities on computed tomography scan (CT-scan) and lymphocytic and eosinophilic alveolitis, with no specific cytologic or microbiological findings, 2 months after pomalidomide initiation.

**Intervention and outcome:**

Antibiotics were started after bronchoscopy. No improvement was noted in dyspnea and biological inflammatory syndrome after 5 days of treatment. Pomalidomide was then discontinued, with continuation of Daratumumab-Dexamethasone, resulting in a rapid recovery of symptoms and CT-scan anomalies. No recurrence of dyspnea was observed during the 15 months of follow-up.

**Diagnoses:**

Pomalidomide-induced lung injury

**Lessons:**

Pomalidomide-induced lung injury is a rare and serious adverse event that can occur early after Pomalidomide introduction. As pomalidomide use is increasing, the identification of drug toxicity as a possible cause of lung injury appears important. We report a rapid recovery of symptoms and CT-scan anomalies after pomalidomide discontinuation.

## 1. Introduction

Pomalidomide is a thalidomide analogue and immunomodulatory imide drug (IMiD). It is used in combination with low-dose dexamethasone for the treatment of patients with relapsed and/or refractory multiple myeloma (MM) who have received at least 2 previous lines of treatment,^[1]^ including Lenalidomide and a proteasome inhibitor.^[2]^ Recently, its indication was extended to adult patients with Acquired Immune Deficiency Syndrome-related Kaposi sarcoma after failure of antiretroviral therapy and Kaposi sarcoma in adult patients who are Human Immunodeficiency Virus-negative.^[3,4]^

Here, we reported a case of pomalidomide-induced lung injury.

## 2. Case presentation

A 72-years-old male patient undergoing treatment for MM was admitted for progressive worsening of dyspnea without fever for 3 weeks.

His past medical history included arterial hypertension treated with Losartan, Hydrochlorothiazide and Atenolol and a MM diagnosed 2 years ago. MM was initially treated with melphalan/prednisone/velcade for 11 months then ixazomib/lenalidomide/dexamethasone for 9 months, both stopped for relapses. Two months before admission, a new MM chemotherapy line including daratumumab/pomalidomide/dexamethasone was started. There was no history of pulmonary disease and the patient had stopped smoking 40 years ago (10 pack-years).

On clinical examination, the patient exhibited dyspnea for any exercise. No cough or chest pain was described. He was apyretic. The auscultation of chest showed normal vesicular sounds without crackles.

**Figure 1.**
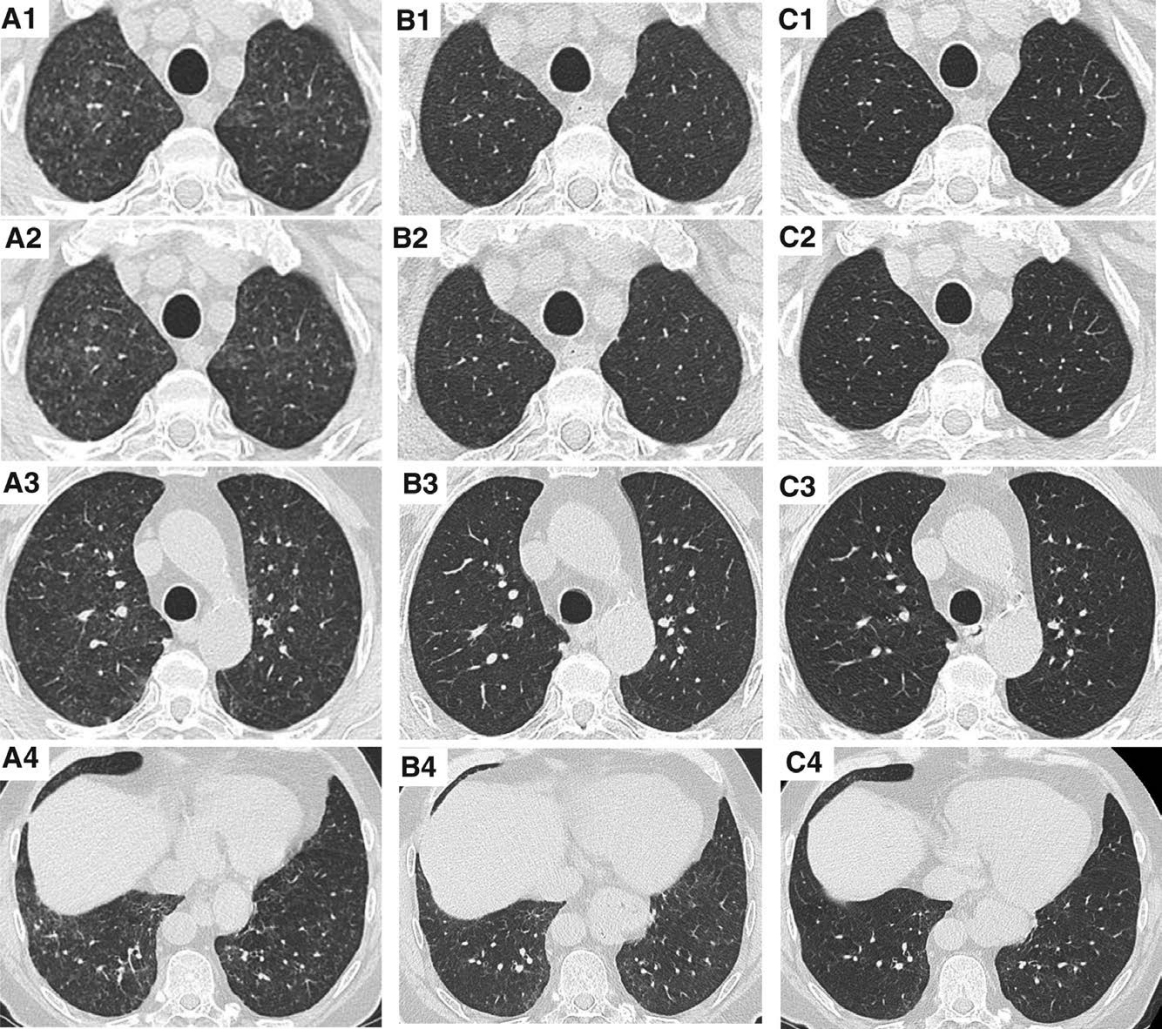
High resolution CT. Chest CT-scan at the initial presentation (*A*_1_, *A*_2_, *A*_3_, *A*_4_), after one month of pomalidomide discontinuation (*B*_1_, *B*_2_, *B*_3_, *B*_4_), and after four months of pomalidomide discontinuation (*C*_1_, *C*_2_, *C*_3_, *C*_4_). CT = computed tomography. CT-scan = computed tomography scan.

Chest computed tomography (CT)-scan showed multiple tiny nodules randomly distributed in both lungs, associated with apical ground glass opacities (Fig. 1A). Laboratory tests revealed an elevation of inflammatory markers (c-reactive protein 45 mg L^–1^) associated to a known stable bicytopenia (white blood cells 0.8 giga L^–1^, hemoglobin 8.2 g dL^–1^). The arterial blood gases showed moderate hypoxemia (room air PaO_2_: 72 mm Hg) and hypocapnia (PaCO_2_: 30 mm Hg). Flexible bronchoscopy was performed before starting any therapeutic intervention. Bronchoalveolar lavage cell count found an elevation of lymphocytes and eosinophils percentage (168,000 cells mL^–1^, 44% lymphocytes, 30.5% macrophages, 25% eosinophils and 0.5% neutrophils) with no specific cytologic or microbiological findings. Pulmonary function tests revealed a low alveolar–capillary membrane diffusing capacity (carbon monoxide diffusing capacity of the lung 37% of predicted value with a restrictive pattern (total lung capacity 5.230 L; 73% of predicted value).

No improvement in dyspnea and biological inflammatory syndrome was obtained after 5 days of antibiotics that were started after bronchoscopy. Because pomalidomide-associated lung injury was suspected, pomalidomide was discontinued, with continuation of daratumumab-dexamethasone. No steroid treatment was introduced. Dyspnea gradually decreased and finally disappeared. One month later, PaO_2_ was normal (room air PaO_2_: 96 mm Hg; PaCO_2_: 34 mm Hg) and chest computed tomography scan (CT-scan) showed disappearance of multiple tiny nodules and of most ground glass opacities (Fig. 1B). At 4 months, carbon monoxide diffusing capacity of the lung also improved (63% of predicted value) with normalization of both total lung capacity (7.690 L; 108% of predicted value) and chest CT-scan (Fig. 1C). Because of the diagnosis of pomalidomide-associated lung injury, pomalidomide was definitely stopped. Pomalidomide, was switched to cyclophosphamide. Under this treatment, his general condition worsened leading to several other treatment strategies. Successive different treatment lines failed to control MM evolution. The patient finally died 15 months after the pulmonary event. There was no recurrence of dyspnea during this medical follow-up.

Because of the retrospective non-interventional design and anonymous management of the data, informed consent of the patient for inclusion was waived, as approved by the French national commission for personal data protection (Comité National de l’Information et des Libertés) (n°2049775 v 0) according to the Jardé law in France.

## 3. Discussion

The mechanisms of action of pomalidomide include angiogenesis inhibition, immunomodulation, impeding cytokine production, and interaction with bone marrow and tumor microenvironment.^[5]^ It interferes with cellular cycle and apoptosis.^[6]^ Most of these mechanisms are common with the other IMiDs derived from thalidomide.^[7,8]^

The major pulmonary adverse event reported with pomalidomide is infectious pneumonia^[9–11]^ (occurs in 15 to 20% of treated patients), related to neutropenia. Thromboembolic events, which are also reported with Lenalidomide and Thalidomide, occur in 1% of patients treated with pomalidomide.^[12]^ Although no evidence of the drug toxicity was highlighted in randomized clinical trials, drug-mediated lung injury related to thalidomide, lenalidomide and pomalidomide have been described in a few case reports, ^[12–15]^.

The 7 case reports^[16–21]^ and the present case report of pomalidomide-induced lung injury are summarized in Table 1. These cases present several similarities. Respiratory symptoms seem to be abrupt or rapidly progressive for most of the cases which is similar to our case report. Mid to upper ground glass lung opacities are the most frequent radiological pattern. Elevated bronchoalveolar lavage lymphocytes and/or eosinophils percentages have been reported, as well as alveolar hemorrhage. Pulmonary infection was ruled out in these cases. Pomalidomide discontinuation was associated in all the cases with a dramatic and rapid improvement, with rare residual pulmonary fibrosis. Only 1 case reported by Gajic^[19]^ showed fibrotic peripheric reticular abnormalities 3 weeks after Pomalidomide discontinuation, with no later CT-scan performed. As the risk of recurrence of lung injury with the reintroduction of pomalidomide seems to be high (3 of the 4 cases previously reported), we decided to not re-use this treatment.

**Table 1:** Summary of published reports on Pomalidomide-induced lung injury and clinical patterns.

Author, yr	Clinical symptoms	Latency from Pomalidomide introduction	CT-scan findings	BAL results	Therapeutic managemenrt t	Outcome
Geyer, 2011^[16]^	-abrupt dyspnea -O_2_ desaturation	480 d	patchy ground glass opacities mid to upper lungs with consolidative characteristics	considered as normal	antibiotics + Pomalidomide discontinuation	-rapid improvement -relapse at reintroduction
Geyer, 2011^[16]^	-abrupt dyspnea -fever -O_2_ desaturation	120 d	patchy ground glass opacities mid to upper lungs with diffuse central lobular fluffy infiltrates	43% macrophages 5% lymphocytes 45% eosinophils	Pomalidomide discontinuation + steroid treatment	-rapid improvement -reintroduction without recurrence
Tello, 2013^[17]^	-respiratory failure -dyspnea -fever	7 d	diffuse alveolar opacities	alveolar hemorrhage	Pomalidomide discontinuation + steroid treatment	-rapid improvement -relapse at reintroduction
Modi, 2015^[18]^	-progressive dyspnea -cough -fever -O2 desaturation	8 mo	ground glass opacities upper lungs	not realized	antibiotics + Pomalidomide discontinuation + steroid treatment	-progressive improvement -relapse at reintroduction
Gajic, 2018^[19]^	-progressive dyspnea -O2 desaturation	22 mo	ground glass opacities mid to upper lungs, fibrosis	30% lymphocytes	Pomalidomide discontinuation + steroid treatment	-progressive resolution -fibrosis lesions
Kumar, 2018^[20]^	-respiratory failure -cough -fever	unknown	consolidation of both lungs, pleural effusion	Not realized	antibiotics + antifungals + Pomalidomide discontinuation	rapid resolution
Icard, 2021^[21]^	-fever -lethargy	6 days	Ground glass opacities, diffuse interlobular thickening	71% lymphocytes	Antibiotics + corticosteroids + Pomalidomide discontinuation	Rapid resolution
2022	-dyspnea	2 mo	Tiny nodules, apical ground glass opacities	44% lymphocytes, 31% macrophages, 25% eosinophils	Antibiotics + Pomalidomide discontinuation	Rapid resolution

BAL = bronchoalveolar lavage, CT-scan = computed tomography scan.

The latency of lung injury onset since the pomalidomide introduction between 6 days to 22 months. In our case, the time exposure to Pomalidomide was relatively short (2 months). This could be explained by an early diagnosis of lung injury, as the clinical involvement and the extent of CT abnormalities were mild in our case. Then, as there is close structural homology between IMiDs, it is conceivable that prior IMiD exposure could prime patient’s immune system for hypersensitivity to Pomalidomide. Our patient was previously exposed to Lenalidomide for 11 months. We note that the reported case with the latest onset of lung injury (22 months) did not have a previous exposure to another IMiD. However, as there are a low number of published reports on pomalidomide-induced lung injury, which do not all report previous dosage and/or duration of exposure to other IMiDs, it is unclear whether prior IMiD exposure could interfere with the time to onset of lung injury. It is also unknown whether lung injury would occur with other IMIDs and would thus be considered a “class effect.” Based on these observations, we suggest that when possible, it is probably safe to avoid other IMiDs use after the occurrence of pomalidomide-induced lung injury.

## 4. Conclusion

Pomalidomide-induced lung injury is an uncommon but serious adverse event that can occur early after Pomalidomide introduction. With increasing use of pomalidomide, recognition of drug toxicity as a possible cause of lung injury is important, particularly given that we noticed a swift reversibility of lesions and symptoms at the pomalidomide discontinuation.

## Author contributions

**Conceptualization:** Alexandre Vivien, Julien Ancel, Sophie Godet, Sandra Dury, Jeanne-Marie Perotin, Gaetan Deslee, Claire Launois.

**Investigation:** Alexandre Vivien, Julien Ancel, Sophie Godet, Sandra Dury, Jeanne-Marie Perotin, Gaetan Deslee, Claire Launois.

**Resources:** Alexandre Vivien, Julien Ancel, Sophie Godet, Sandra Dury, Jeanne-Marie Perotin, Gaetan Deslee, Claire Launois.

**Visualization:** Alexandre Vivien, Julien Ancel, Sophie Godet, Sandra Dury, Jeanne-Marie Perotin, Gaetan Deslee, Claire Launois.

**Writing – original draft:** Alexandre Vivien, Julien Ancel, Sophie Godet, Sandra Dury, Jeanne-Marie Perotin, Gaetan Deslee, Claire Launois.

**Writing – review & editing:** Alexandre Vivien, Julien Ancel, Sophie Godet, Sandra Dury, Jeanne-Marie Perotin, Gaetan Deslee, Claire Launois.
